# Response of *Carex breviculmis* to phosphorus deficiency and drought stress

**DOI:** 10.3389/fpls.2023.1203924

**Published:** 2023-07-11

**Authors:** Songlin Jiang, Yiqing Tang, Rong Fan, Shidong Bai, Xiaoqi Wang, Yulin Huang, Weizhong Li, Wenli Ji

**Affiliations:** ^1^ College of Landscape Architecture and Art, Northwest A&F University, Yangling, China; ^2^ College of Forestry, Northwest A&F University, Yangling, China

**Keywords:** Carex breviculmis, phosphorus deficiency, drought, trait, ecological strategy

## Abstract

**Introduction:**

The drought and phosphorus deficiency have inevitably become environmental issues globally in the future. The analysis of plants functional trait variation and response strategies under the stress of phosphorus deficiency and drought is important to explore their ability to respond to potential ecological stress.

**Methods:**

In this study, *Carex breviculmis* was selected as the research object, and a 14-week pot experiment was conducted in a greenhouse, with two phosphorus treatment (add 0.5mmol/L or 0.05μmol/L phosphorus) and four drought treatment (add 0-5%PEG6000), totaling eight treatments. Biomass allocation characteristics, leaf anatomical characteristics, biochemical parameters, root morphology, chemical element content, and photosynthetic parameters were measured.

**Results:**

The results showed that the anatomical characteristics, chemical elements, and photosynthetic parameters of *Carex breviculmis* responded more significantly to main effect of phosphorus deficiency. Stomatal width, leaf phosphorus content and maximum net photosynthetic rate decreased by 11.38%, 59.39%, 38.18% significantly (*p*<0.05), while the change in biomass was not significant (*p*>0.05). Biomass allocation characteristics and root morphology responded more significantly to main effect of drought. Severe drought significantly decreased leaf fresh weight by 61% and increased root shoot ratio by 223.3% compared to the control group (*p*<0.05). The combined effect of severe drought and phosphorus deficiency produced the highest leaf N/P ratio (291.1% of the control) and MDA concentration (243.6% of the control). Correlation analysis and redundancy analysis showed that the contributions of phosphorus and drought to functional trait variation were similar. Lower epidermal cell thickness was positively correlated with maximum net photosynthetic rate, leaf phosphorus, chlorophyll ab, and leaf fresh weight (*p*<0.05).

**Discussion:**

In terms of response strategy, *Carex breviculmis* was affected at the microscopic level under phosphorus deficiency stress, but could maintain the aboveground and underground biomass well through a series of mechanisms. When affected by drought, it adopted the strategy of reducing leaf yield and improving root efficiency to maintain life activities. *Carex breviculmis* could maintain its traits well under low phosphorus and moderate drought, or better conditions. So it may have good ecological service potential in corresponding areas if promoted. This study also provided a reference for plant response to combined drought and phosphorus deficiency stresses.

## 1 Introduction

Currently, the global environment is facing various issues. Exploring the ecological strategies of plants in response to environmental stress is a hot topic in plant ecology and plant application research (Moeneclaey et al., 2022; Yu et al., 2022).

The soil around global is faced with the dilemma of depletion of phosphorus, which is a non-renewable resource. The available chemical form of phosphorus that can be directly utilized by plants in the soil is very scarce due to the mineralization of organic phosphorus and the easy conversion of inorganic phosphorus into an unusable form (Zhou et al., 2017). The ecological strategies of some plants in response to low phosphorus stress have practical value, as they can ensure the plants growth and contribute to ecological restoration and alleviation of soil degradation. The ecological strategies are worth researching and promoting in low-phosphorus areas (Muler et al., 2014).

In terms of climate, many regions of the world are trending towards drought (Han et al., 2021; Gessner et al., 2022). Drought stress has a significant impact on plant survival and yield, which further affects ecological environment and socio-economic stability (Schimpl et al., 2019; Aguiar et al., 2020). However, the response mechanisms and strategies of plants to drought are extremely complex and diverse. Therefore, in ecological planning, the selected plants need to have excellent ecological strategies for possible drought climates, in order to construct sustainable plant communities proactively (Bastin et al., 2019).

To study the ecological strategies of plants in response to phosphorus and drought stress, quantitative research on plant traits is an important methodology. Plant traits cover many aspects, including yield distribution characteristics and morphological characteristics of various organs, anatomical traits, and nutrient element content indicators (Flexas et al., 2022). These trait indicators have different responses to the environment: some indicators have a synergistic relationship, and some have a reverse change. It reflects the trade-off strategy of plants which put limited resources proportionally into different levels of different trait in responce to environmental stress (Wright et al., 2004). For example, traits associated with faster growth rates (acquisitive strategy) often related to larger and thinner leaves with shorter lifespans, while those involving considerable resource storing (conservative strategy) related to the opposite. They represent the extremes of an ecological continuum. In addition, some researchers believe that analyzing the ecological strategies of plants more comprehensively requires measuring traits at multiple levels, including biomass allocation, morphology, physiology, and other aspects (Crainejoseph, 2009; Freschet et al., 2018).

Plants respond to drought stress by exhibiting a decrease in leaf photosynthetic rate, reduction in biomass, and an increase in root shoot ratio. Tabassum investigated 113 horticultural species and employed trait-based approaches to identify three differential trait-investment strategies that plants adopt when facing drought stress. Plants exposed to drought stress exhibit negative responses in leaf traits such as leaf fresh weight and the ratio of leaf fresh weight to dry weight (Tabassum et al., 2021). Carvajal ‘s research showed that plants of the same species employ different trait-investment strategies to cope with drought stress due to differences in the aridity gradient of their environments (Carvajal et al., 2017).

Plants exhibit various strategies to cope with phosphorus deficiency, including morphological, biochemical, and physiological responses, ultimately enabling them to survive better under low-phosphorus conditions (Yuan and Liu, 2008).Researcher Wen found a trade-off between phosphorus (P) acquisition strategies among 16 crops: species with finer roots tend to alter root morphological traits to acquire P, while species with thicker roots increase mycorrhizal colonization or release more root exudates containing carboxylates. Li’s research demonstrated that readily available phosphorus was one of the main factors affecting multiple leaf traits in desert herbaceous plants. Zhao’s study showed that low-P treatment increased Caragana’s leaf mass per area (Meier et al., 2023) and leaf dry matter content (LDMC), and had a negative impact on photosynthetic capacity (Zhao et al., 2013).

However, it is not fully understood how plants respond to drought and phosphorus deficiency at the same time, which are likely to be more widespread in the future climate. Some scholars investigated the negative effects of different drought treatments on plant nitrogen and phosphorus uptake (He and Dijkstra, 2014; Suriyagoda et al., 2014). Josipovic believed that phosphorus application can reduce the damage of drought to yield to a certain extent (Josipovi et al., 2012). Namugwanya used bean development as an example to study agricultural countermeasures to cope with phosphorus deficiency and drought stress in soil (Namugwanya et al., 2018).They generally believed that drought and phosphorus deficiency will become important constraints in future habitats. The drought may affect phosphorus uptake and transport, exacerbating negative effects on plant production. However, these studies mainly focus on individual key traits and fail to analyze the trade-offs among multiple plant traits. It is not conducive to evaluate the response strategies adopted by plants, which makes it difficult to measure their value for ecological application value in the future.

It is necessary to analyze the relationship between various plant traits and environmental stress, and evaluate whether plants have good coping strategies (Bastin et al., 2019). After identifying potential plants with stress tolerance, social managers, botanists, and seedling producers need to jointly carry out advanced ecological planning (Zölch et al., 2018). These potential plants can be promoted gradually in the wild environment that require ecological restoration and in residential area that require low maintenance costs. Ultimately, it will provide more stable ecological service capability with lower management costs in the future (Li et al., 2021).

Carex are a promising object for studying plant traits in response to phosphorus deficiency and drought stress. Plants of the Carex family are distributed across multiple continents and climate zones and appear in low-phosphorus and drought-prone regions, occupying advantageous ecological niches and demonstrating strong adaptability and ecological restoration potential (Playsted et al., 2006; Masuda et al., 2021). As one of the most widely used plants in this family, *Carex breviculmis* is known to have drought-tolerant physiological characteristics (Y. Wang et al., 2023) and is valued for its beautiful form and color in landscaping (Mi et al., 2022). Overall, *Carex breviculmis* has potential in ecological restoration and landscape design.

In this study, we used *Carex breviculmis* as the research object and set up cross-stress conditions of P and drought to explore the multi-faceted trait responses of *Carex breviculmis*, such as biomass allocation, morphology, physiology, and chemical element content, and attempt to answer the following questions: (1) Clarify the impact of multiple drought and phosphorus deficiency treatments on plant functional traits. (2) Explore the synergistic and trade-off relationships among *Carex breviculmis* traits. (3) Evaluate the *Carex breviculmis*’s response strategies to drought and P deficiency stress.

This study analyzed the ability of *Carex breviculmis* to resist drought and low phosphorus, providing a reference for studying plant trait changes and corresponding strategies under compound stress. Plants with better coping strategies can be involved in future ecological planning, cultivated and produced in advance, and applied in areas where the climate and environment are deteriorating. This project is especially important in degraded natural environments, and low-maintenance plant landscapes in urban areas.

## 2 Experimental materials and methods

### 2.1 Experimental materials and process

The plant material used in this study was *Carex breviculmis* R. Br., collected from a university campus in Shanxi Province, China (E108.075288, N34.266719), and stored in a greenhouse. The temperature was maintained at 24-32°C, and artificial light was used as a supplement to natural light during cloudy weather. The central wavelength of artificial light was 589nm, the red blue ratio was 8.5:1, the luminous flux was 48000lm, the height was 2 meters, the horizontal spacing was 2.5 meters, and the light–dark cycle was 16/8 (light/darkness, hours).

The experiment began in May 2022 and was conducted for 14 weeks. *Carex breviculmis* were carefully uprooted from the original substrate with minimal damage to the root system and rinsed clean. They were then uniformly trimmed to a height of about 12cm above ground and a root length of about 10cm, with a total weight of 2g. Each plant was transplanted individually into a 13cm diameter pot. A mixture of high-purity quartz sand of different particle sizes, totaling 850g as the substrate (Φ0.3 mm: Φ0.7 mm: Φ2mm = 7:2:1, m/m/m). The pot’s position was randomly changed every two weeks to eliminate environmental influences. Deionized water was used to water the plants for the first two weeks of the experiment to deplete plant’s mineral nutrients (Ji et al., 2020). From the third week onwards, nutrient solution was applied according to the treatment conditions. The plants were watered once a week with a nutrient solution of 100ml each time. The nutrient concentration increased each week, starting at 1/4, then to 1/2, 3/4, and finally full concentration. The nutrient solution formula used Hoagland’s method (Hoagland and Arnon, 1950).

The experiment was conducted using a completely randomized design, including two levels of phosphorus treatment and four levels of drought treatment, for a total of eight treatment levels. The phosphorus treatment varied in the content of KH_2_PO_4_ (high phosphorus concentration: 0.5 mmol/L, represent by HP; low phosphorus concentration: 0.05 μmol/L, represent by LP). The drought treatment varied in the concentration (w/w) of added PEG6000, and whether or not the weighing method was used for watering. According to the degree of drought from strong to weak, four treatment conditions were represented by ABCD in sequence. A: Add 5% PEG6000.B: Add 2.5% PEG6000. C: Do not add PEG6000. D: Do not add PEG6000 and supply water according to weighing method. The weighing method was to supplement 100ml of deionized water when the water content is below 40% of the saturated water content. The details and abbreviations of all treatment conditions were provided in **Table 1**. Among them, HPD was the control group (high phosphorus concentration, no PEG6000, watered timely), which was considered a relatively ideal treatment for plant growth. Each treatment was replicated six times, and at the start of the experiment, there were a total of 48 samples. At the end of the experiment, three of them that had survived were randomly selected for data collection.

**Table 1:** Schematic table of experimental treatments.

Treatment group	Phosphorus treatment	Drought treatment
HPA	Add 0.5m mol/L phosphorus	Add 5% PEG6000
HPB	Add 0.5m mol/L phosphorus	Add 2.5% PEG6000
HPC	Add 0.5m mol/L phosphorus	Do not add PEG6000
HPD	Add 0.5m mol/L phosphorus	Do not add PEG6000 and supply water according to weighing method.
LPA	Add 0.05μmol/L phosphorus	Add 5% PEG6000
LPB	Add 0.05μmol/L phosphorus	Add 2.5% PEG6000
LPC	Add 0.05μmol/L phosphorus	Do not add PEG6000
LPD	Add 0.05μmol/L phosphorus	Do not add PEG6000 and supply water according to weighing method.

### 2.2 Sampling and measurement

#### 2.2.1 Measurement of photosynthetic parameters

Photosynthetic parameters were collected using a portable photosynthesis system, LI-6400 (LI-COR, Lincoln, Nebraska, USA). Three days before harvest, photosynthetic parameters were measured using an automated light response curve measurement program between 08:00-11:30 and 15:00-18:00. Photosynthetically Active Radiation (PAR) of photosynthesis was set as: 1500, 1200, 1000, 750, 500, 250, 200, 150, 100, 50, 25, 0 μmol m^−2^s^-1^, flow rate set 500 μmol *s^-1^ and concentration of CO_2_ set 400 μmol CO_2_ *mol^-1^, Tblock 25°C (Busch and Lösch, 1998). Results of the light response curve were imported into the software, Photosynthesis, to calculate the light saturation point, maximum net photosynthetic rate, and dark respiration rate.

#### 2.2.2 Measurement of leaf biomass and specific leaf area

After plant harvest, leaves and roots were separated. Use analytical balance for weighing. The required amount of leaves was weighed for subsequent biochemical analysis and anatomical measurements. Three to five mature leaves were selected. The leaves’ projected area was obtained using the scanner Winrhizo and dried weight was weighed to calculate SLA, the ratio of leaf area to leaf dry weight (cm^2^/g) (Lienin and Kleyer, 2011). The remaining leaves were dried and weighed to determine the leaf dry matter content (LDMC).

#### 2.2.3 Measurement of biochemical parameters

Chlorophyll content was extracted from fresh leaves of *Carex breviculmis* using 95% (v/v) ethanol. The absorbance value was measured at 665, 649, and 470 nm to calculate the content of chlorophyll a and b (Lichtenthaler and Wellburn, 1983). The concentration of malondialdehyde (MDA) and soluble sugars (SS) was determined using the thiobarbituric acid method at 532, 600, and 450 nm (Weng et al., 2015).

#### 2.2.4 Measurement of leaf anatomical characteristics

Leaf sections were dehydrated in a graded series of ethanol solutions, infiltrated with warm paraffin (56-58°C), dried, and sectioned. The sections were stained with methylene blue solution and observed under a light microscope to measure length (Liu et al., 2021). The following indicators were recorded: stomatal length and width, leaf thickness, bubble cell thickness.

#### 2.2.5 Measurement of root biomass and root morphology

The harvested fresh roots were weighed. The root system images were obtained using the Winrhizo scanner, and root morphology indices were analyzed. Then dried the root and weighed. Some root index was calculated as follows: specific root length (SRL, m*g^−1^, the ratio of root length to root dry mass), root tissue density (RTD, g*cm^−3^, root dry mass divided by fresh root volume), the ratio of root surface area to root dry biomass (RSAB, cm^2^*g^−1^), branching intensity (BI, cm^−1^, ratio of branch number to root length) (Kang et al., 2022).

#### 2.2.6 Measurement of chemical element content

The roots and leaves of the plants were separated, weighed, washed with deionized water, dried, and ground for chemical analysis. Carbon content was determined by the potassium dichromate wet-oxidation method, nitrogen content was determined by the indophenol blue colorimetric method, and phosphorus content was determined by the vanadium molybdate yellow colorimetric method (Zhang et al., 2021).

### 2.3 Statistical analysis method

All data were tested for normality using the Shapiro-Wilke test and for homogeneity of variance using the Levene test. If the results don’t obey the normal distribution, the logarithmic function and the square root function are adopted to form normal distribution. Fisher’s LSD was used to analyze the statistical significance of the differences between the different treatments (*p*<0.05). Factorial analysis was used to judge the effects of phosphorus and drought on each index. Spearman correlation was used to analyze the relationship between each plant functional traits. Redundancy analysis (RDA) was used to study the relationship between the experimental conditions and the traits after logarithmic processing.

## 3 Result

### 3.1 Photosynthetic parameter results

According to the results of the light response curve, software analysis was used to obtain the maximum light saturation point, maximum photosynthetic value, and dark respiration rate, as shown in **Table 2**. The maximum light saturation point and maximum photosynthetic value of the low phosphorus treatment group were 37.0% and 38.2%, lower than those of the high phosphorus treatment group, and both differences were significant (*p*<0.05). The light saturation point of HPD reached 816μmol·m^-2^·s^-1^ and maximum photosynthetic rate reached 9.452μmol·m^-2^·s^-1^. These values were significantly higher than most other treatment groups (*p*<0.05).

**Table 2:** Photosynthetic parameter results.

Treatment group	Light saturation point/μmol·m^-2^·s^-1^	Maximum net photosynthetic rate/μmol·m^-2^·s^-1^	Dark respiration rate/μmol·m^-2^·s^-1^
LPA	413 ± 103.034 bc	4.843 ± 1.047 cd	-0.383 ± 0.054 abc
LPB	446 ± 41.737 bc	4.833 ± 0.338 cd	-0.429 ± 0.086 ab
LPC	409 ± 129.275 bc	5.008 ± 0.853 bcd	-0.318 ± 0.039 bc
LPD	284 ± 111.83 c	4.334 ± 0.94 1d	-0.257 ± 0.136 bc
HPA	587 ± 207.519 ab	7.546 ± 2.573 abc	-0.433 ± 0.024 ab
HPB	413 ± 20.396 bc	6.113 ± 0.872 bcd	-0.200 ± 0.177 c
HPC	649 ± 37.974 ab	7.653 ± 0.893 ab	-0.550 ± 0.091 a
HPD	816 ± 153.46 a	9.452 ± 1.721 a	-0.432 ± 0.083 ab

Mean ± SE, N=3. Different letters indicate significant differences in mean values between treatments based on ANOVA analysis.

### 3.2 Biomass allocation characteristics and specific leaf area results

**Figure 1:**
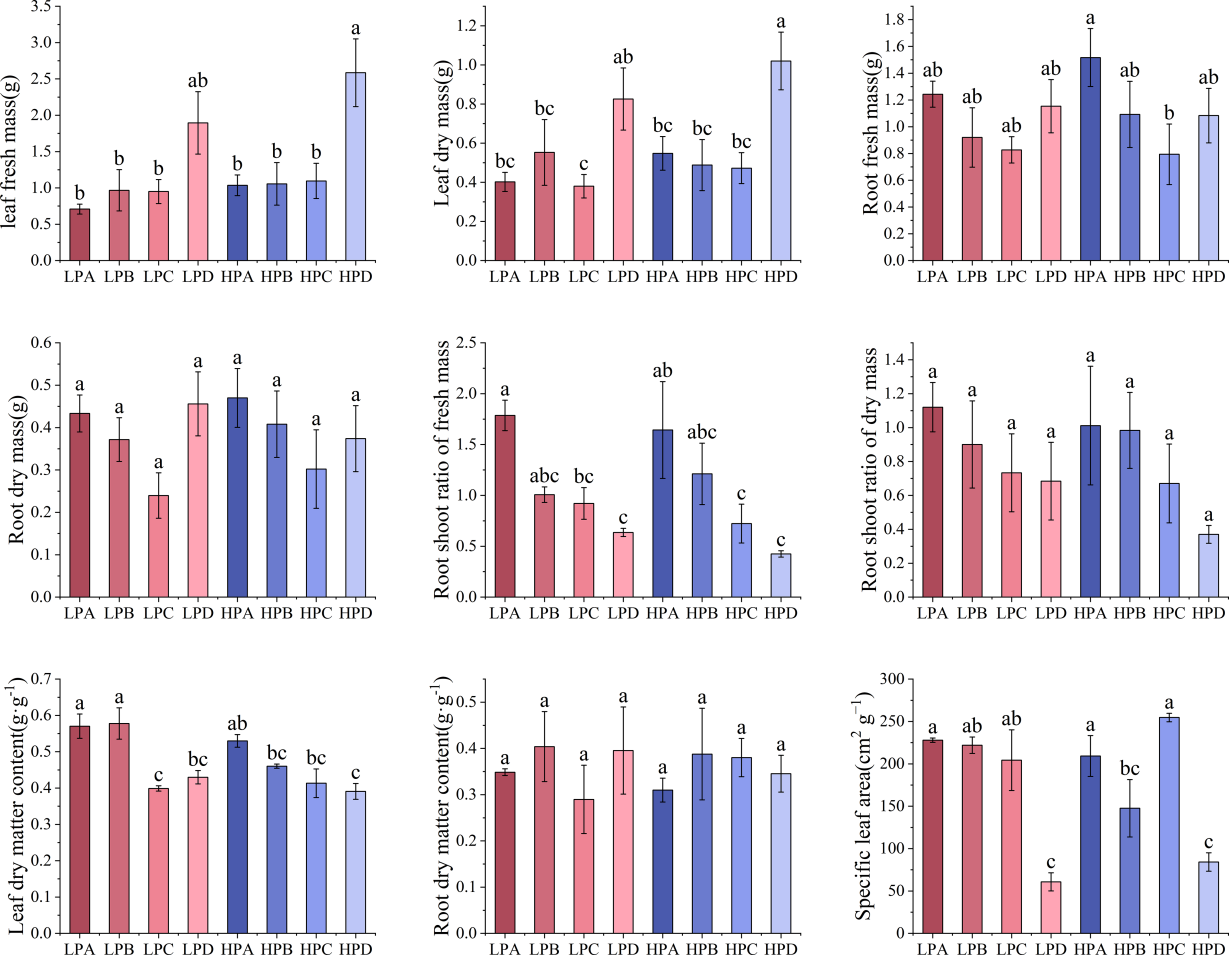
The effect of phosphorus and drought treatments on the biomass allocation (Mean ± SE, N=3). Different letters indicate significant differences in mean values between treatments based on ANOVA analysis.

**Figure 1** showed the biomass allocation characteristics and specific leaf area results. The results of fresh weight and dry weight of leaves showed that in both high-phosphorus and low-phosphorus treatments, drought treatment D group led to the highest values. Leaf fresh weight was 1.895g in LPD, 2.586g in HPD, and values from 0.708g to 1.096g in other treatment groups. The value of the HPD group is the highest and was significantly higher than all other groups (*p*<0.05). The difference in leaf fresh weight between high and low phosphorus groups was not significant (P>0.05).

The results of fresh weight of roots showed that in both high- and low-phosphorus group, drought treatment A led to the relative highest value of 1.517g, while treatment C had the lowest value under drought treatment of 0.794g. The differences between most of the treatment values were not significant. The differences in dry weight of roots were not significant.

The results of root shoot ratio of fresh weight showed that in both high- and low-phosphorus treatments, the values increased monotonically as the degree of drought treatment increased. Within the high- and low-phosphorus treatments, treatment A led to the highest value which was significantly higher than those of C and D groups (*p*<0.05). In high phosphorus treatment, the HPA value was 1.464, which is 3.494 times higher than HPD. In low phosphorus treatment, the LPA value was 1.755, which is 2.885 times that of LPD.

The results of leaf dry matter content showed that in both high- and low-phosphorus treatments, the values of drought treatment A are all higher than 0.5 and significantly higher than treatment D (*p*<0.05). There were no significant differences in root water content between the treatments.

The results of specific leaf area showed that in both high- and low-phosphorus treatments, the values of treatment D were the lowest. In the high-phosphorus treatment, the values of HPA, HPB, and HPC ranged from 147.588 cm^2^*g^-1^ to 254.700 cm^2^*g^-1^, while the HPD value were 84.163 cm^2^*g^-1^, significantly lower than other three treatments (*p*<0.05). In the low-phosphorus treatment, the values of LPA, LPB, and LPC ranged from 204.360 cm^2^*g^-1^ to 227.970 cm^2^*g^-1^, while the HPD values were 60.713 cm^2^*g^-1^, significantly lower than other three treatments (*p*<0.05).

### 3.3 Leaf biochemical parameters results

**Figure 2:**
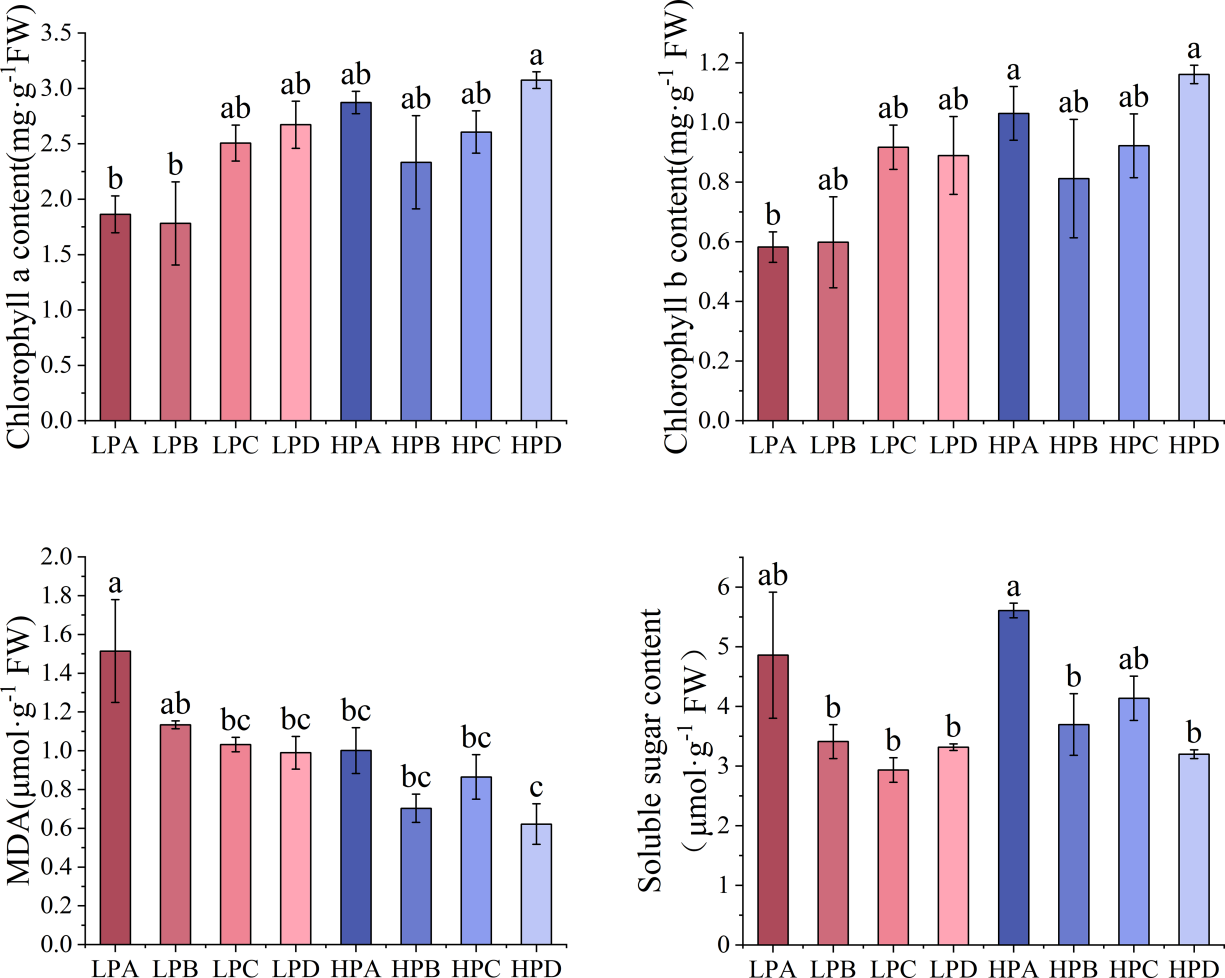
The effect of phosphorus and drought treatments on the concentration of biochemical parameters in leaves (Mean ± SE, N=3). Different letters indicate significant differences in mean values between treatments based on ANOVA analysis.

The results of leaf biochemical parameters were shown in **Figure 2**. The results of chlorophyll a showed that in both high- and low-phosphorus treatments, treatment D had the highest values, but the differences were not significant compared to the other drought treatments. Among all treatments, the HPD had the highest value of 3.074mg*g^-1^FW, significantly higher than the LPA(1.864 mg*g^-1^FW) and LPB(1.781 mg*g^-1^FW) (*p*<0.05). The results of chlorophyll b showed that the HPD had the highest result, 1.161 mg * g^-1^FW, significantly higher than the LPA (*p*<0.05), and the differences between the other treatments were mostly not significant.

The results of MDA showed that the LP treatment was higher than the HP treatment overall. In the LP treatment, the LPA value was 1.514μmol *g^-1^ FW, significantly higher than most treatment groups, and they ranged from 0.621 to 1.134μmol *g^-1^ FW (*p*<0.05). In the HP treatment, the HPD value was 0.621μmol *g^-1^ FW, which was the relatively lowest value. The results of soluble sugar showed that in both high- and low-phosphorus treatments, treatment A had the highest value of 5.609μmol *g^-1^ FW, with HPA significantly higher than many other treatment (*p*<0.05).

### 3.4 Leaf anatomy indicators results

**Figure 3:**
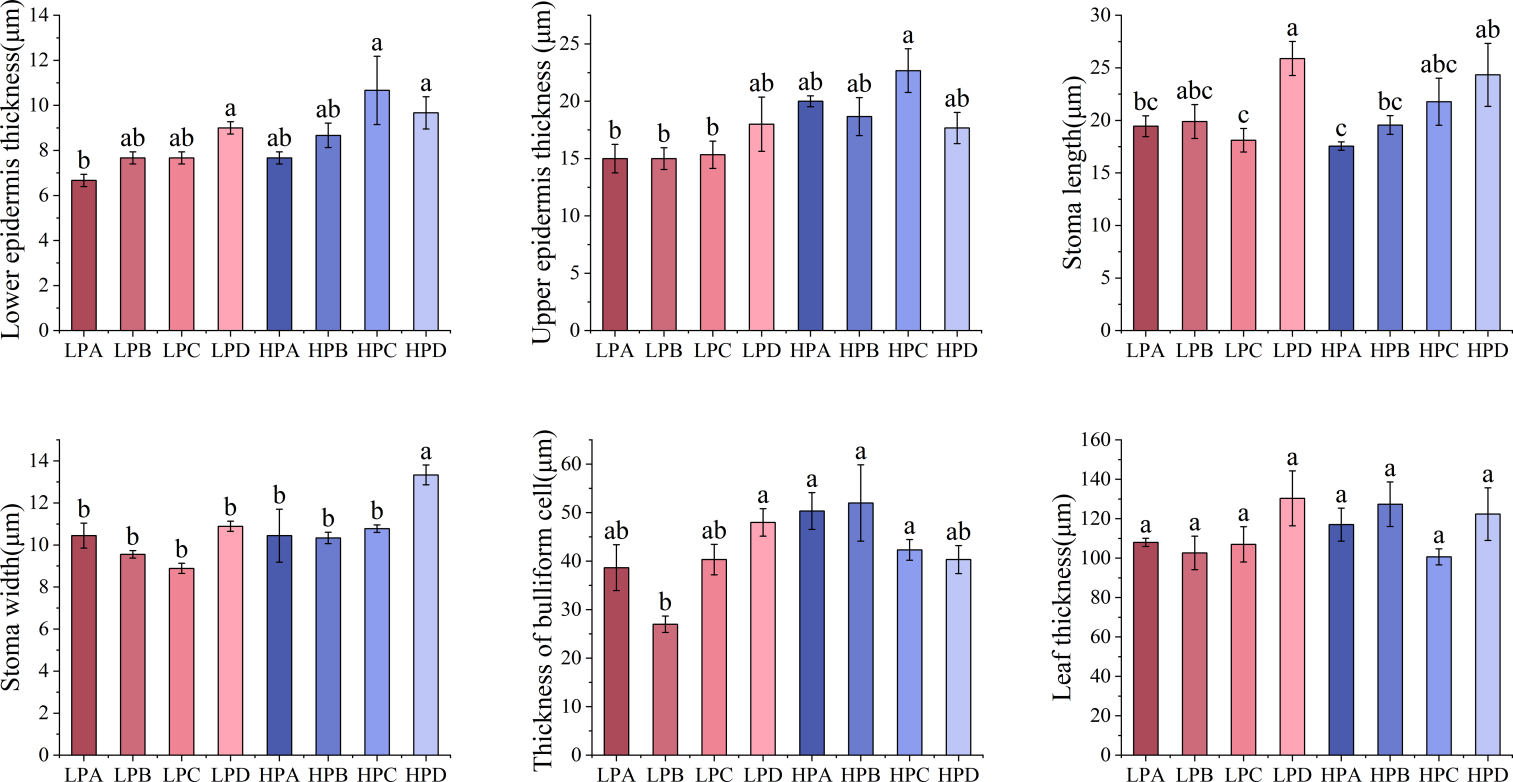
The effect of phosphorus and drought treatments on the leaf anatomy indicator. Different letters indicate significant differences in treatment means based on ANOVA analysis. The data of epidermal thickness in each treatment were transformed to conform to normal distribution in ANOVA analysis.

**Figure 3** showed that epidermal thickness values in the high phosphorus treatment were generally higher than those in the low phosphorus treatment. The highest Stomatal length value was found in the HPD (24.333μm) and LPD (25.889μm) (*p*<0.05), the values for other treatments ranged from 17.556μm to 19.889μm. The stomatal width of the low phosphorus group is 11.38% lower than that of the high phosphorus group (p<0.05). Stomatal width in the HPD group was 13.333μm significantly higher(*p*<0.05) than in other (ranged from 8.889μm to 10.889μm).

### 3.5 Chemical element content results

**Figure 4:**
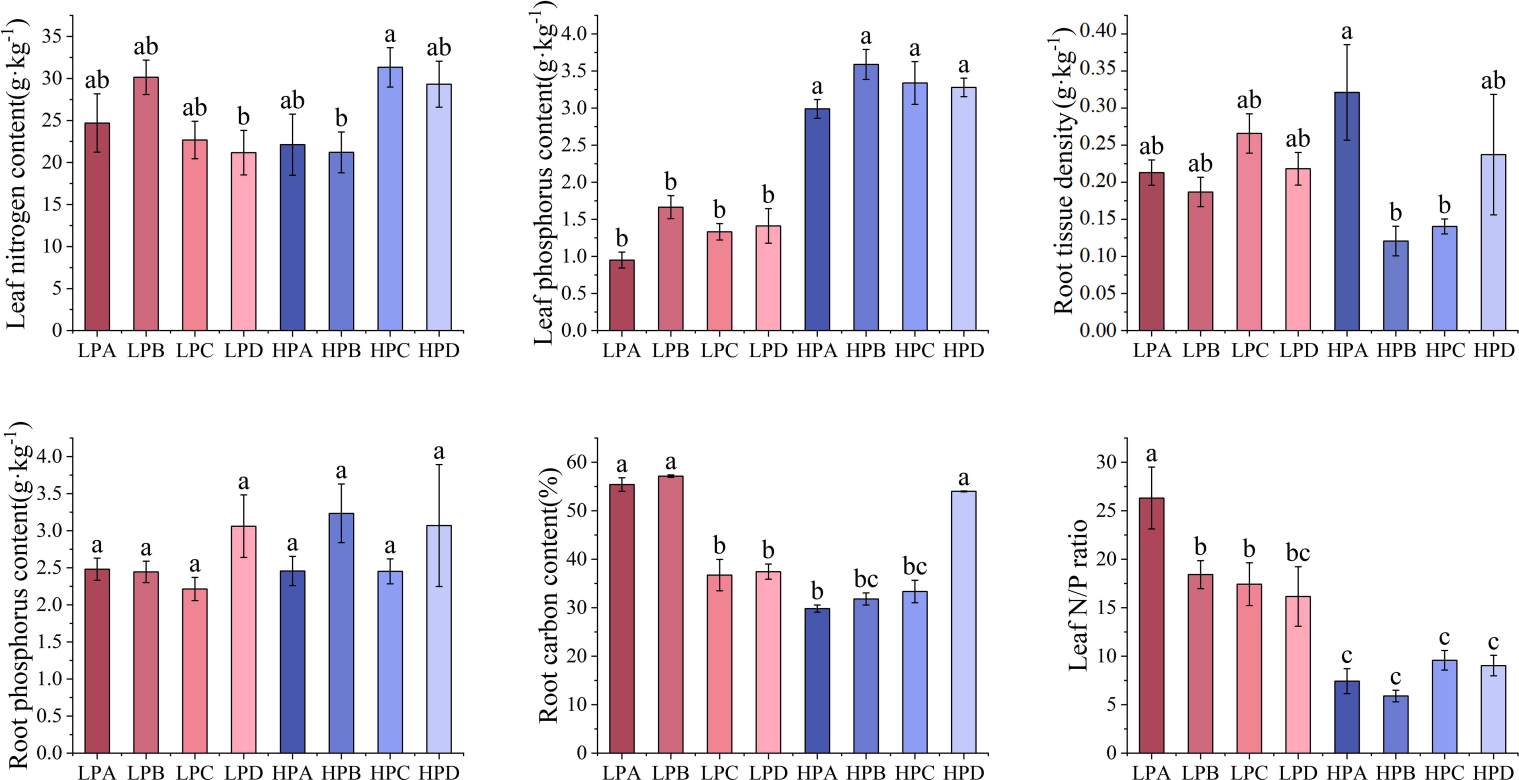
The effect of phosphorus and drought treatments on the chemical element content. Different letters indicate significant differences in treatment means based on ANOVA analysis.

As shown in **Figure 4**, leaf phosphorus content in the high phosphorus treatment ranged from 0.951g*kg^-1^ to 1.664g*kg^-1^, which was significantly higher than 2.990g*kg^-1^to 3.590g*kg^-1^ in the low phosphorus treatment (*p*<0.05). The leaf phosphorus content of the low phosphorus group decreased by 56% compared to the high phosphorus group. But differences among the drought treatments were not significant. Differences in leaf nitrogen and root phosphorus content among treatments were mostly not significant. Root nitrogen content was highest in the HPC treatment (21.182g*kg^-1^), and differences among the other treatments were mostly not significant.

Root carbon content in the LPA, LPB, and HPD ranged from 54.011% to 57.150%, which was significantly higher than the 29.820% to 37.451% of other treatments (*p*<0.05). Leaf nitrogen to phosphorus ratio of the low phosphorus treatment ranged from 16.161 to 26.314, mostly higher than 5.896 to 9.588 for all high phosphorus treatments. Leaf nitrogen to phosphorus ratio of LPA was 26.314, significantly higher than all other treatments.

### 3.6 Root morphology results

**Figure 5:**
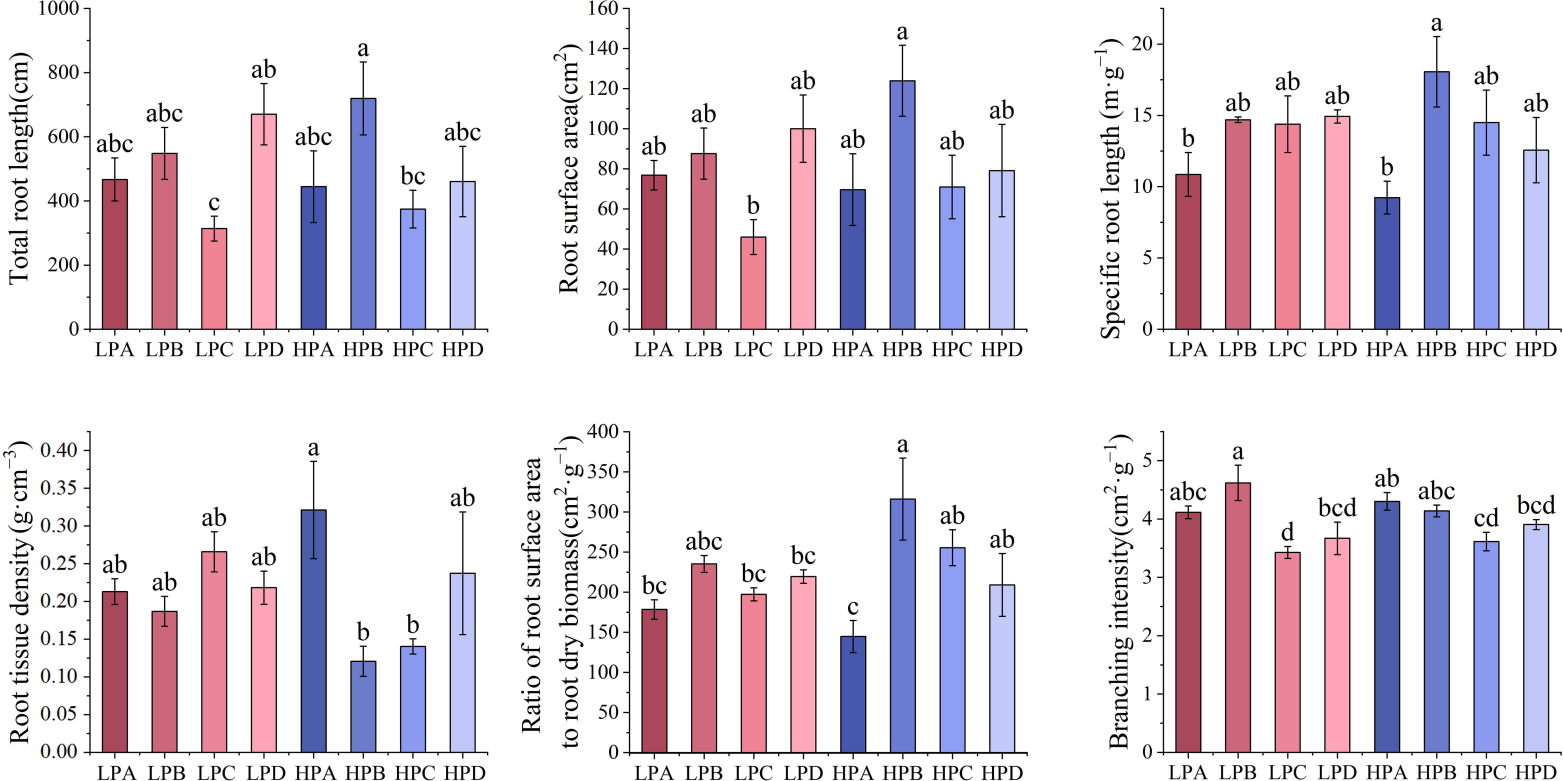
The effect of phosphorus and drought stress on the root morphology. Different letters indicate significant differences in treatment means based on ANOVA analysis.

Results of root morphology are shown in the **Figure 5**. The highest total root length was 719.786cm in the HPB and lowest was 313.857cm in the LPC. The results of surface area showed that the highest value in the high phosphorus treatment was 123.958cm^2^ in HPB, and the highest in the low phosphorus treatment was LPD value of 100.056cm^2^. In all treatments, the HPB had the highest value, and was significantly higher than the LPC (*p*<0.05), while differences among the other treatments were not significant.

According to the results, the A treatment had the lowest values in both the high phosphorus and low phosphorus groups for root length. The specific root length of HPA (10.857m*g^-1^) and LPA (9.235 m*g^-1^) was the lowest, and the other six treatments ranged from 12.564m*g^-1^ to 18.065m*g^-1^. The root tissue density was the relatively highest in the HPA, and there is no significant difference between other groups. The ratio of root surface area to root dry biomass was lowest in HPA (144.774cm^2^*g^-1^) and highest in HPB (316.221cm^2^*g^-1^). The results of the branching number to root length ratio showed the highest value for LPB and the lowest value for LPC.

### 3.7 Factorial analysis results

The results of the factorial analysis (**Table 3**) showed that the primary effects of phosphorus deficiency stress on most biomass distribution characteristics indicators and root morphology indicators were not significant. However, leaf fresh and dry weight, root shoot ratio of fresh weight, leaf dry matter content, specific leaf area, root surface area, root dry weight, branch intensity all had significant main effects under drought stress (*p*<0.05), indicating that drought had a significant impact on these indicators. Among them, F values of leaf dry matter content and specific leaf area were the highest, which were both over 10, indicating that the drought treatment had the strongest effect on them. Furthermore, there was a significant interactive effect between phosphorus deficiency stress and drought stress on leaf dry matter content (*p*<0.05).

**Table 3:** The effects of phosphorus deficiency and drought stress on various traits based on factorial analysis.

	Trait		Main effect of phosphorus deficiency stress		Main effect of drought stress			Interaction between phosphorus deficiency and drought		
		F	*p*	Partial eta square	F	*p*	partial eta square	F	*p*	partial eta square
Biomass distribution characteristics	Leaf fresh mass	1.540	0.233	0.088	6.420	0.005*	0.546	0.289	0.833	0.051
	Root fresh mass	0.254	0.621	0.016	1.955	0.161	0.268	0.234	0.871	0.042
	Leaf dry mass	0.235	0.635	0.014	6.161	0.005*	0.536	1.296	0.310	0.196
	Root dry mass	0.490	0.827	0.003	1.690	0.209	0.241	0.289	0.833	0.051
	Root shoot ratio (fresh mass)	0.196	0.664	0.556	6.670	0.004*	0.556	0.253	0.858	0.045
	Root shoot ratio (dry mass)	0.256	0.620	0.016	1.473	0.259	0.216	0.170	0.915	0.031
	Root dry matter content	0.147	0.706	0.009	0.475	0.704	0.082	0.614	0.616	0.103
	Leaf dry matter content	4.420	0.052	0.216	10.266	0.001*	0.658	3.426	0.043*	0.391
Sla and srl	Specific leaf area	0.033	0.858	0.002	16.627	0.000***	0.757	2.363	0.110	0.307
	Specific root length	0.007	0.935	0.000	3.062	0.580	0.365	0.709	0.561	0.117
Root morphology	Root tissue density	0.210	0.653	0.013	1.829	0.182	0.255	2.107	0.140	0.283
	Ratio of root surface area to root dry biomass	1.106	0.309	0.447	4.311	0.021*	0.447	1.457	0.264	0.215
	Branching intensity	0.044	0.837	0.003	6.326	0.005*	0.543	1.218	0.335	0.186
	Total root length	0.001	1.000	0.340	2.753	0.077	0.340	1.106	0.376	0.172
	Root surface area	0.371	0.551	0.023	2.242	0.123	0.296	0.965	0.433	0.153
Leaf anatomy indicators	Upper epidermis cell thickness	9.166	0.008*	0.364	0.491	0.693	0.084	1.542	0.242	0.224
	Lower epidermis cell thickness	5.255	0.036*	0.247	2.636	0.085	0.331	0.745	0.541	0.123
	Stomatal length	0.000	0.985	0.000	4.094	0.025*	0.434	0.776	0.524	0.127
	Stomatal width	7.197	0.016*	0.310	4.884	0.013*	0.478	1.329	0.300	0.199
	Bubble cell thickness	4.848	0.043*	0.233	0.459	0.714	0.079	3.926	0.028*	0.424
	Leaf thickness	0.335	0.571	0.020	1.233	0.330	0.188	0.836	0.494	0.136
Chemical element content	Leaf nitrogen content	0.307	0.587	0.019	0.390	0.762	0.068	3.250	0.050*	0.379
	Root nitrogen content	2.368	0.143	0.129	0.670	0.583	0.112	2.885	0.068	0.351
	Root carbon content	42.473	0.000***	0.726	10.975	0.000***	0.673	48.723	0.000***	0.901
	Root phosphorus content	0.598	0.451	0.036	1.052	0.397	0.165	0.329	0.805	0.058
	Leaf phosphorus content	158.439	0.000***	0.908	2.990	0.062	0.359	0.063	0.979	0.012
	Leaf N/P ratio	46.728	0.000***	0.745	1.585	0.232	0.229	2.548	0.092	0.323
Leaf biochemical parameters	Mda	12.119	0.003*	0.431	3.344	0.046*	0.385	0.495	0.691	0.085
	Chla	3.851	0.067	0.194	1.670	0.213	0.238	0.560	0.649	0.095
	Chlb	3.471	0.081	0.178	1.132	0.366	0.175	1.103	0.377	0.171
	Soluble sugar	1.843	0.193	0.103	4.845	0.014*	0.476	0.520	0.674	0.089
Photosynthetic parameter	Maximum net photosynthetic rate	19.765	0.000***	0.553	0.782	0.521	0.128	1.460	0.263	0.215
	Dark respiration rate	0.920	0.352	0.054	0.647	0.596	0.108	0.174	0.912	0.032
	Light saturation point	15.149	0.001*	0.486	0.804	0.510	0.131	3.964	0.027*	0.426

* indicates a significant correlation(p<0.05),*** indicates a highly significant correlation(p<0.05),

Regarding anatomical indicators, the thickness of upper and lower epidermal cells and stomatal width were significantly affected by phosphorus deficiency stress (*p*<0.05), while stomatal length was significantly affected by drought stress (*p*<0.05). The thickness of spongy cells was significantly affected by the main effects of phosphorus deficiency stress and the interaction effects of phosphorus and drought stress (*p*<0.05).

Chemical element content analysis showed that the effects of phosphorus deficiency stress were significant on root carbon content, leaf phosphorus content, and leaf nitrogen-to-phosphorus ratio (*p*<0.05). The effect of drought stress was significant on root carbon content, while there was a significant interactive effect on leaf nitrogen and root carbon (*p*<0.05).

Results of biochemical parameter analysis showed that the main effects of drought and phosphorus deficiency stress were significant on malondialdehyde (MDA) (*p*<0.05). The F value of the drought treatment reached 12.119, which was nearly three times higher than the F value of the phosphorus deficiency treatment. It might indicate that drought caused stronger oxidative stress than phosphorus deficiency.The effect of drought stress was significant on soluble sugar content (*p*<0.05).

Photosynthetic parameters showed that the primary effects of phosphorus deficiency stress were significant on maximum photosynthetic value and light saturation point (*p*<0.05). Their F values all exceeded 15, indicating a strong effect of phosphorus deficiency on photosynthetic parameters. The interactive effect on light saturation point was significant (*p*<0.05).

Simple effect analysis was conducted on indicators which were affected by significant interactive effects. The simple effect of phosphorus treatment was significant (*p*<0.05) on leaf dry matter content and spongy cell thickness only under drought treatment C. Similarly, the simple effect of phosphorus treatment was significant (*p*<0.05) on root carbon content only under drought treatment ACD. Finally, the simple effect of phosphorus treatment was significant (*p*<0.05) on light saturation point only under drought treatment A.

### 3.8 Correlation analysis

**Figure 6:**
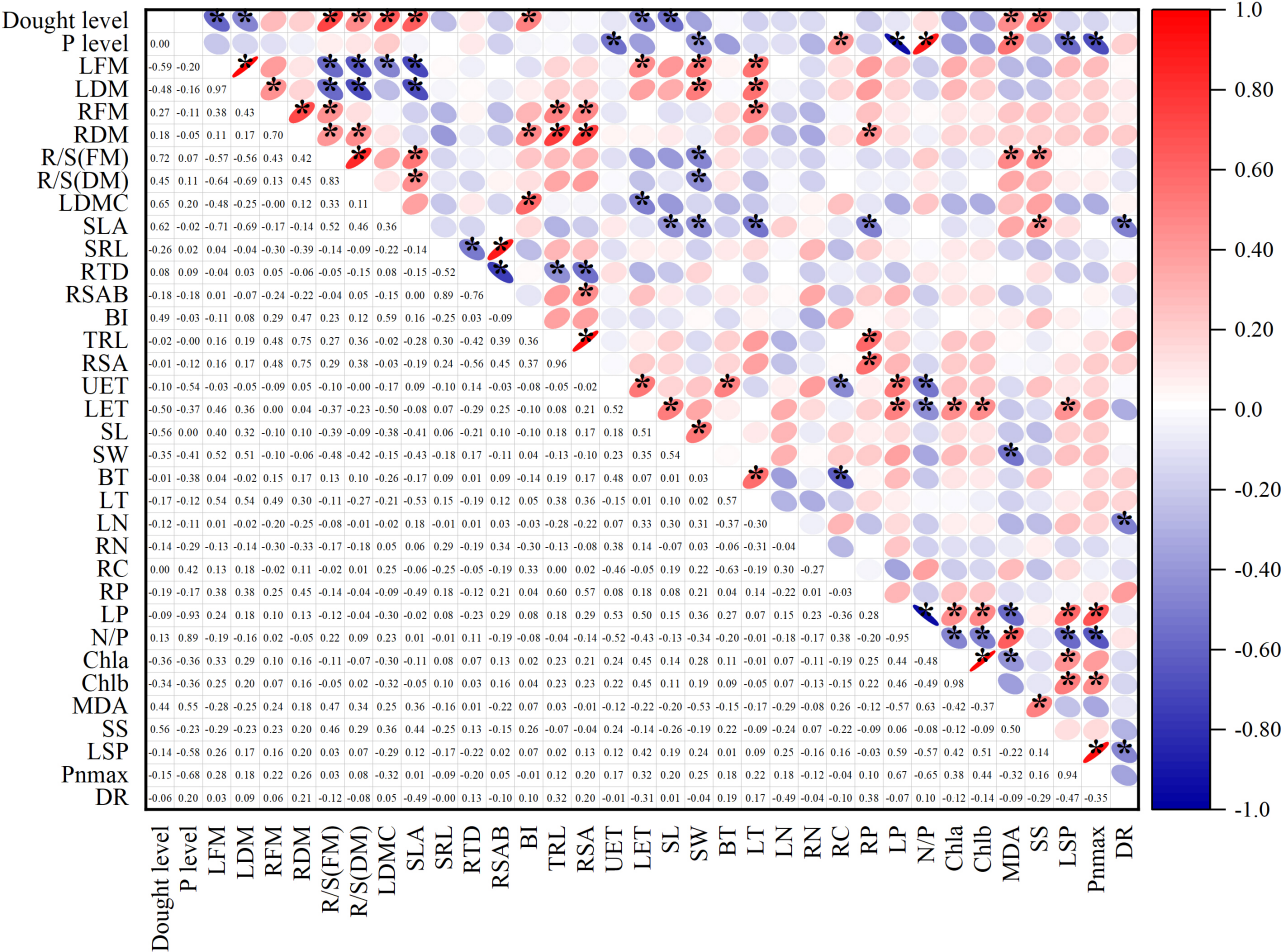
Spearman correlation analysis showed the relationship between treatment levels and functional traits. In the area in the lower left corner, the number represents the correlation coefficient. In the upper right corner, blue indicates a negative correlation, red indicates a positive correlation, and * indicates a significant correlation. Interpretation of abbreviations: LFM, Leaf fresh mass; LDM, Leaf dry mass; RFM, Root fresh mass; RDM, Root dry mass; R/S(FM), Root shoot ratio (fresh mass); R/S(DM), Root shoot ratio (dry mass); LDMC, Leaf dry matter content; SLA, Specific leaf area; LN, Leaf nitrogen content per mass; LP, Leaf phosphorus content per mass; RN, Root nitrogen content per mass; RP, Root phosphorus content per mass; RC, Root carbon content per mass; SRL, Specific root length; RTD, Root tissue density; RSAB, the Ratio of root surface area to root dry biomass; BI, Branching intensity; TRL, Total root length; RSA, Root surface area; UET, Upper epidermis cell thickness; LET, Lower epidermis thickness; SL, Stomatal length; SW, Stomatal width; BT, Bubble cell thickness; LT, Leaf thickness; N/P, Leaf N/P ratio; Chla, chlorophyll a; Chlb, chlorophyll b; MDA, malondialdehyde concentration; SS, Soluble sugar concentration; LSP, light saturation point, Pnmax-maximum net photosynthetic rate; DR, dark respiration rate.

The Spearman correlation analysis showed the relationship between the treatment levels and functional traits. In the **Figure 6**, the numbers in the lower left corner represent the correlation coefficients. In the upper right corner, blue indicates negative correlation, red indicates positive correlation, and an asterisk indicates a significant correlation. The following only showed results with significant correlation (*p*<0.05).

The correlation analysis to some extent indicates the co-variation relationship between various indicators. Among them, leaf fresh weight was positively correlated with stomatal length and width, leaf thickness, and negatively correlated with leaf dry matter content. Root fresh weight was positively correlated with root shoot ratio, root length, and other root morphology indicators, and negatively correlated with root dry matter content. Specific leaf area was positively correlated with root shoot ratio, leaf dry matter content, and soluble sugar content, and negatively correlated with total biomass, fresh and dry leaf weight, root phosphorus content, stomatal length and width, leaf thickness, and respiration rate. Root carbon content was positively correlated with leaf nitrogen-phosphorus ratio. Root phosphorus content was positively correlated with biomass and root weight. Leaf phosphorus was positively correlated with light saturation point, maximum photosynthetic value, chlorophyll a and b, carotenoids, upper and lower epidermal cell thickness, and negatively correlated with MDA. Among the anatomical indicators, the thickness of the lower epidermis was related to multiple types of indicators, positively related to leaf phosphorus, fresh leaf weight, leaf dry matter content, photosynthetic pigments, and light saturation point. Root indicators had a few significant correlations with other types of indicators. The absolute values of correlation coefficients between different types of traits are mostly between 0.4 and 0.6.

### 3.9 Redundancy analysis

Redundancy analysis (RDA) was performed on 16 functional trait indicators that could comprehensively represent the various aspects of the research material and had a good response to the experimental treatments. These indicators were selected based on the results of causality and correlation analyses, and indicators with poor response and those directly derived from basic indicators were excluded (Flexas et al., 2022).

**Figure 7:**
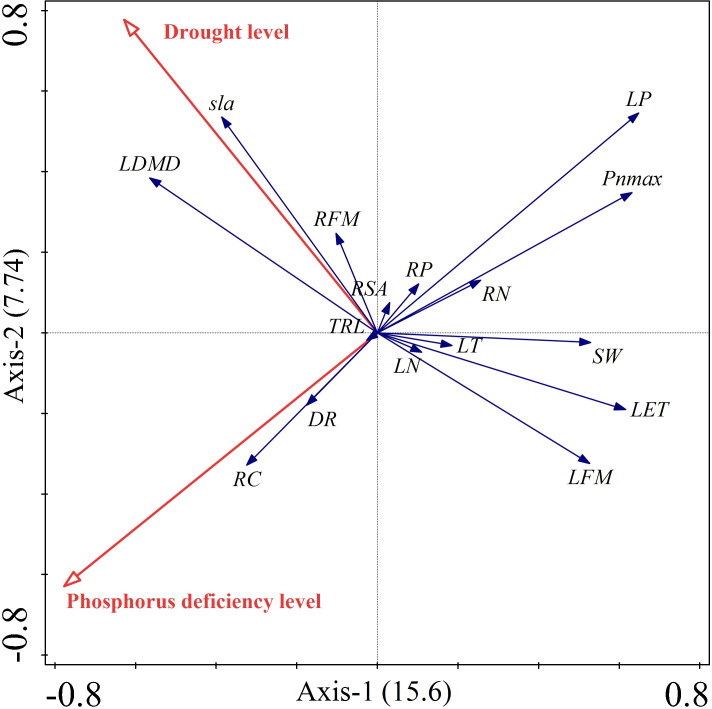
Dual diagram of RDA. Red vector arrows represent the conditioning factors, and blue vector arrows represent the response variables.

**Supplementary Tables 1**, **2** showed that both phosphorus deficiency and drought significantly contributed to the traits of *Carex breviculmis*. Phosphorus deficiency contributed 53.5% while drought contributed 46.5% to the trait variation, indicating a similar contribution from both treatments. The RDA biplot displayed the treatment factors and plant indicators as two-dimensional arrows, where the length and direction of the arrows reflected the treatment conditions’ influence on the plant indicators, and the balance of the indicators was reflected by the arrows between them (**Figure 7**). The biplot indicated an orthogonal relationship between drought and phosphorus deficiency stresses. Drought was strongly positively correlated with leaf dry matter content, specific leaf area, and root fresh weight, but negatively correlated with leaf weight and anatomical traits. Phosphorus deficiency was positively correlated with root carbon content and dark respiration rate, but negatively correlated with leaf phosphorus content, root nitrogen content, maximum photosynthetic rate, stomatal width, lower epidermal thickness, and leaf fresh weight. Leaf phosphorus was negatively correlated with root carbon, while leaf dry matter content was negatively correlated with leaf fresh weight. Anatomical traits were positively correlated with leaf fresh weight and leaf phosphorus, and maximum photosynthetic rate. These results were consistent with the results of correlation analysis.

## 4 Discussion

### 4.1 Response of *Carex breviculmis* to phosphorus deficiency stress

Overall, phosphorus deficiency had a minor effect on biomass allocation characteristics, root morphology, and root elemental content. However, it had a significant impact on leaf anatomical indicators, leaf chemical element content, and photosynthesis.

Leaves are the most adaptable and plastic organs to environmental changes, and can clearly reflect the effects of various stresses on plant growth and development. In this experiment, there was a large difference in phosphorus concentration between the two treatment levels, but the response of each yield allocation index to phosphorus deficiency stress was not significant. Some researchers believed that some of the plants with conservative growth strategies reduced their growth rate, nitrogen metabolism and utilization efficiency by adjusting their internal nitrogen utilization strategy under phosphorus limitation, thereby maintaining biomass (Moeneclaey et al., 2022; Han et al., 2021). This may explain why *Carex breviculmis* maintained its yield under phosphorus deficiency stress, but the specific mechanism needed further study. This characteristic made *Carex breviculmis* had the potential to be applied in phosphorus-deficient soils, thereby maintaining the biomass of ground cover plants with basic ecological service capacity.

At the same time, phosphorus deficiency had a significant main effect on leaf anatomical indicators, including upper and lower epidermal thickness and stomatal width. Previous studies have shown that phosphorus concentration can affect the proportion of primary meristem tissues in plants and the input of photosynthetic-related tissues, and cell elongation may also be limited by low phosphorus availability (Corrêa et al., 2017). These results may explain why some leaf tissue cells at low phosphorus levels have smaller microscopic sizes.

The impact of phosphorus deficiency was reflected directly in the chemical element content of leaves, with significant main effects on leaf phosphorus content, leaf nitrogen-phosphorus ratio. Due to phosphorus deficiency, plants would reduce the transport of leaf phosphorus and regulated the proportion of metabolic phosphorus to ultimately reduce the phosphorus content of the leaves (Tao et al., 2022; Yu et al., 2022). At the same time, phosphorus levels significantly affected the relationship between plant nitrogen and phosphorus. The leaf N:P ratio was generally regarded as an indicator of plant soil nutrient limitation. A ratio greater than 16 indicated phosphorus limitation. Some also believed that leaf N/P ratio reflected the balance between N and P absorption and loss, and the adjustment of helping plants better responded to the availability of nutrients in phosphorus-deficient soils (Drenovsky and Richards, 2004). According to the results, the nitrogen-phosphorus ratios of all drought treatments in the low phosphorus treatment group were greater than 16, indicating the phosphorus limitation of *Carex breviculmis*. At the same time, the nitrogen-phosphorus ratio of the A treatment (5% PEG6000) was significantly higher than that of other drought treatments, indicating that heavy drought stress exacerbates phosphorus limitation. Some studies have suggested that drought may exacerbate phosphorus deficiency symptoms, possibly due to osmotic pressure affecting the flow of phosphate ions, leading to a decrease in plant root absorption efficiency (Suriyagoda et al., 2014), or drought affecting root microbiota and enzyme activity related to root phosphatase and dehydrogenase. Another possible reason was that further affecting phosphorus absorption and transport (Paz-Ares et al., 2022).

Phosphorus deficiency had a significant negative impact on photosynthetic parameter, reducing the light saturation point and maximum net photosynthetic rate. Some researchers believed that low phosphorus supply and low leaf phosphorus might reduce the photosynthetic capacity of leaves due to the reduction in proteins that regulate CO_2_ enrichment, the Calvin cycle and the electron transport system (Thomas et al., 2006). However, *Carex breviculmis* maintained biomass despite the reduced photosynthetic productivity, which could be attributed to the improved efficiency of phosphorus recycling in senescent tissues or the deceleration of cellular metabolism (Han et al., 2021).

### 4.2 Response of *Carex breviculmis* to drought stress

In response to drought stress, *Carex breviculmis* exhibited changes in biomass allocation, root morphology, and partial leaf anatomical indices, while its chemical elements content and photosynthetic performance showed no significant alterations.

Leaves are the primary organs for plant transpiration, and more leaf biomass means greater water consumption, while roots are the primary organs for water absorption. Thus, under drought stress, plants adjust the allocation of above- and below-ground biomass to optimize water use and maintain their vital functions (Loudari et al., 2022).

Drought stress had a significant impact on biomass allocation features. Among the high and low phosphorus groups, the value of leaf weight in the non-drought stress treatment group was the highest, while the HPD (high phosphorus non drought treatment group) value was the highest among the eight treatment groups, significantly higher than most stress treatment groups. This result was consistent with previous studies that have verified the negative effects of drought on plant productivity from the perspectives of photosynthesis, ion transport, and stress resistance mechanisms (Osakabe et al., 2014). According to the results of the factorial analysis, the mild drought treatment C already significantly affected the fresh and dry weights of leaves but did not further decrease them as the degree of drought stress increased. Drought stress also leaded to an increase in leaf dry matter content, which was a direct consequence of reduced water content, but it also indicated that plants increased the solute content inside their bodies to enhance osmotic pressure and reduce water loss through transpiration (Barros et al., 2020).

In addition to the direct effect on leaf biomass, drought stress significantly affected the allocation proportion of resources between roots and leaves. The root shoot ratio is a sensitive growth index in plant stress physiology, which reflects the adjustment of plant biomass allocation to improve the absorption of light, water, and nutrients. In this study, the response of the root shoot ratio to phosphorus deficiency stress was not significant, but the response to drought stress was very clear, manifested by a significant decrease in leaf biomass while root biomass was almost unaffected. The numerical value of the root shoot ratio decreased from large to small according to the intensity of drought treatment (ABCD). This was consistent with previous research results, indicating the drought response strategy which *Carex breviculmis* reduced the resource investment in leaves and maintained the resource investmennt in roots on the one hand, this strategy reduced water evaporation caused by leaves, and on the other hand, it maintained the investment in roots to ensure the plants’ ability to obtain water from the substrate (Luo et al., 2021).

In terms of leaf anatomy indicators, both the length and width of stomata in *Carex breviculmis* responded significantly to drought stress. The length and width of stomata in the non-drought treatment (D) were both significantly higher than those in the drought-treated group (ABC treatments). Previous studies have shown that the shrinkage of stomata can reduce water transpiration loss, while also affecting carboxylation efficiency and sucrose phosphate synthase activity, leading to a decrease in plant productivity (Yang et al., 2022). This is also corroborated by the decrease in leaf biomass. On the other hand, drought reduced cell turgor pressure, which caused damage to cell division and inhibits elongation and extension. This leads to in the size of some cells, such as stomatal cells, being affected (Santos et al., 2015; Khan et al., 2023).

The impact of drought on the functional characteristics of root morphology was also significant, including root surface area and root dry biomass, the branch intensity, etc. When water supply in the substrate was insufficient, plants changed their root morphology to improve nutrient acquisition efficiency and improve their own growth, including biomass, root absorption area, number and length of root tips, etc. These indicators reflected that the root system of *Carex breviculmis* was using as few resources as possible, increasing the number of branches and the contact area between roots and soil, and further improving the efficiency and cost-effectiveness of resource absorption by the root system (Kang et al., 2022).

### 4.3 Dual effects of phosphorus and drought stress

The results of the dual effects of phosphorus deficiency and drought stress were noteworthy. Among the high and low phosphorus treatments, Treatment B (2.5% PEG application) had the highest ratio of root surface area to root dry biomass and the highest branching intensity. This might indicate that moderated drought stress, relative to no drought stress and severe drought stress, may induce plants to adopt a more effective strategy for resource acquisition by the root system. Additionally, treatment B in the high phosphorus treatment had the highest values for total root length and root surface area among all treatments, and was higher than Treatment B in the low phosphorus treatment. When the phosphorus supply was sufficient, plants might also invest more in the root system to cope with other stresses, such as drought, some studies have also confirmed this phenomenon (Jing et al., 2010).

Some studies have shown that fertilization can partially alleviate the damage caused by drought stress. The results of chlorophyll and MDA content in this study were consistent with this view, especially for Treatment A under drought stress. The high phosphorus treatment had a healthier value than the low phosphorus treatment (Xia et al., 2020).

The results of the redundancy analysis indicated that the functional trait response of *Carex breviculmis* was jointly affected by phosphorus and water. The light saturation point results showed that the phosphorus treatment has a significant simple effect (*p*<0.05) only under the A drought treatment. It was evident that phosphorus content significantly affected the level of light saturation point during severe drought stress. Previous studies have shown that phosphorus is an important element involved in photosynthesis, while drought reduced the important substrate of photosynthetic reactions, which was water (Wang et al., 2022). Under the dual stress of severe drought and phosphorus deficiency, the saturation point of photosynthetic reactions was lower, indicating that this indicator was more severely affected than under other treatments. At the same time, as mentioned earlier, the nitrogen to phosphorus ratio was significantly higher in the LPA treatment than in all other treatments. It may indicate that severe drought treatment exacerbates phosphorus deficiency symptoms in *Carex breviculmis*.

### 4.4 Trade-offs and synergies among different traits

#### 4.4.1 Relationships and response strategies among traits

Plants often acquire limited resources, and how to allocate and utilize these resources reflects their “wisdom”. Exploring the relationships among different plant traits under different experimental conditions can effectively measure the plant’s resource allocation strategies (Yu et al., 2022).

The redundancy analysis and correlation analysis results provided some explanation about the effects of treatment conditions on the measured indicators. The selected indicators for the redundancy analysis covered root and leaf biomass allocation characteristics, leaf anatomical traits, photosynthetic indicators, and root morphology, and both drought and phosphorus deficiency have similar overall contributions to these indicators.

Phosphorus deficiency was positively correlated with root carbon content and dark respiratory rate, while negatively correlated with leaf phosphorus, maximum photosynthetic rate, stomatal width, and epidermal thickness. Phosphorus participated in photosynthesis and cell construction in plants, and its deficiency leaded to a decrease in photosynthetic capacity and affects photosynthetic and respiratory organs.

Drought levels were significantly correlated with several traits. Leaf fresh weight, lower epidermal thickness, stomatal length was negatively correlated with drought levels, while root shoot ratio of dry weight, leaf dry-to-fresh weight ratio, specific leaf area, and branching intensity were positively correlated with drought levels. According to with the previous analysis, it could be concluded that drought had a negative impact on the yield and microstructure of leaves in *Carex breviculmis*. The significant change in leaf dry matter content was also very typical to indicate the stress of drought on leaf physiology (Lienin & Kleyer, 2011). However, the relationship between specific leaf area and drought stress was not consistent with some studies and would be discussed later. At the same time, drought had a positive impact on the biomass proportion and resource utilization efficiency of the root system, which was consistent with previous research.

In the correlation results of various functional traits between leaves and roots, leaf fresh weight was negatively correlated with root shoot ratio, indicating that *Carex breviculmis* had a trade-off between leaf and root investment, especially in drought treatments. The change in root shoot ratio showed a significant decrease in leaf investment to ensure root investment strategy.

The correlation results between leaf biomass and leaf anatomical indices showed that leaf fresh weight was positively correlated with lower epidermal thickness, stomatal width, and leaf thickness, while these anatomical indices were negatively correlated with drought stress levels. This indicated that when drought stress was severe, leaf fresh weight decreased while microstructural size also decreased in coordination to counteract the stress.

It was worth noting that stomatal width and lower epidermal thickness were two representative anatomical indices that were correlated with many key measurement indicators. For example, lower epidermal thickness was positively correlated with fresh leaf weight, stomatal length, and light saturation point, while it was negatively correlated with specific leaf area and root shoot ratio. This finding might link microstructural size with the yield and distribution characteristics, photosynthetic capacity, and chemical element content of plant individuals. Previous studies have shown that the physiological changes in plants in different environments were coordinated with changes in microstructure (Ivanova et al., 2018). The co-evolution of physiological function and leaf anatomical characteristics enhanced the ability of plants to cope with the environment. It confirmed that the thickness of the epidermal cells was significantly positively correlated with local precipitation (Liu et al., 2021). Therefore, some anatomical indices were potential predictable in the trait set.

Root traits, in comparison to leaf traits, were relatively weak predictors. Although some root traits have been shown to improve efficiency under moderate drought stress in Section 4.3, most root traits and their relationships with other root traits or leaf traits were not significantly correlated. This phenomenon was common in the theory of root economics spectrum. Several studies have suggested that root environments were more complex than leaf environments, and root traits exhibited diverse and varied responses to environmental nutrients and stress. Therefore, the responses of root system traits to environmental factors were relatively independent among different species and environments, lacking coordination (Shen et al., 2022). Most root traits, including SRL and RSAB, which were considered to have the potential to become core traits, were not always coordinated with leaf traits. This might be due to mismatches in trait measurement standards or differences in the way plants allocate above-ground and below-ground resources. In summary, the correlations between root traits were difficult to explain clearly (Kramer-Walter et al., 2016).

#### 4.4.2 Discussion of unusual results

Specific leaf area (SLA) was a core indicator of leaf economics spectrum, closely related to multiple leaf traits, and reflected the strategies that plants adopted to cope with ecological environments at the individual level. However, the changes in SLA in this study were not entirely consistent with previous research. Generally, a higher value of SLA often indicated a larger leaf area, thinner leaves, and more exposure to light, all of which reflected higher indicators of leaf resource utilization efficiency of the same quality (Wright et al., 2004; Lienin and Kleyer, 2011). In this study, drought stress caused an increase in the value of SLA in *Carex breviculmis*, and a higher SLA predicted a higher root shoot ratio, wider stomatal length, thicker leaves, and higher dark respiration rates. This reflected that *Carex breviculmis* is more inclined to respond to drought by shrinking stomata and reducing respiration intensity, which is consistent with most studies.

However, some results were not consistent with previous studies on leaf economic spectrum, such as Problem A: the nitrogen-phosphorus correlation between SLA and leaves was not significant, Problem B: higher SLA appeared in drought treatment. Problem C: the changes in SLA and leaf dry matter content were positively correlated.

Problem A may be related to phenology and plant age. In leaf phenology studies, some researchers suggested that leaf traits may be significantly affected by phenology and plant age (Liu et al., 2019). Changes in phenology and plant age may decouple intraspecific trait-trait relationships from the global leaf economics spectrum. Due to the different levels of nitrogen and phosphorus consumption in different leaf stages, the time and size of nitrogen and phosphorus fluxed entering and leaving the leaves differ, resulting in differences in the relationship between chemical elements and traits such as SLA (Townsend et al., 2007; Liu et al., 2021). This made the correlation between SLA and leaf nitrogen and phosphorus elements, especially in mature leaves, may not be significant. In this study, leaf samples were not distinguished by different developmental stages. It was possible that the difference in leaf age and phenology might have led to a decrease in the coordination strength between SLA and nitrogen and phosphorus. Ultimately, this results in their relationship not being consistent with the results of some leaf economics spectrum studies.

The reason for Problem B might be complex. Moderate drought forced plants to adopt a resource gain strategy that maximizes short-term high-resource returns under drought stress, to compensate for losses and cope with risks (Navarro and Hidalgo-Triana, 2021). In addition, previous research had shown that drought led to a down-regulation of sucrose-phosphate synthase activity, which inhibited carbohydrate synthesis, while an up-regulation of invertase promoted the decomposition of related carbohydrates, ultimately reducing carbohydrate content and biomass under drought stress (Li et al., 2023). Meanwhile, plants used the fixed carbon from photosynthesis to produce secondary metabolites to resist drought stress (Attarzadeh et al., 2020). The results of this study indicated that drought had a negative impact on the aboveground biomass and carbon content of rhizomes, while the strategy adopted by *Carex breviculmis* included reducing water loss and increasing specific leaf area, i.e. reducing stomatal and respiratory rated to avoid water evaporation on the one hand, and using fewer leaves, lower leaf mass, and thinner leaf thickness to increase specific leaf area on the other hand, to obtain the largest possible supplement of photosynthetic resources. The response of leaf thickness to drought was considered complex and controversial (Poorter et al., 2009). Therefore, the expansion of leaf area to receive light and compensate for decreased carbohydrate productivity under drought treatment in *Carex breviculmis* might be a response to this unfavorable situation.

Problem C exhibited a rare phenomenon of coordinated changes in SLA and leaf dry matter content, which was supported by a few desert plants. This indicated that the utilization and adjustment ability of leaf tissue in *Carex breviculmis* under drought stress was extremely powerful (Akram et al., 2022). In addition, some studies had shown that the relationship between SLA and LDMC under drought stress might not necessarily be in harmony with environmental gradients (Yang et al., 2022). This warranted further research at the genetic and molecular level in the future.

## 5 Conclusion

In this study, we selected phosphorus deficiency and drought, which may become future environmental stress, as treatment factors, and investigated the response of different traits and the trade-offs between traits in *Carex breviculmis*.

This study showed that, (1) the response of *Carex breviculmis* traits to the two stresses: variations in biomass allocation characteristics and root morphology were mainly caused by drought stress, especially the decrease in leaf weight and the increase in root efficiency. While variations in anatomical traits, chemical elements, and photosynthetic parameters were mainly caused by phosphorus deficiency stress, especially stomatal width, leaf phosphorus content and maximum net photosynthetic rate have decreased. (2) The ecological response strategies of *Carex breviculmis* were as follows: under drought stress, *Carex breviculmis* maintained the balance of physiological functions by reducing the investment of leaf biomass and improving root efficiency. Although some microstructures and physiological functions were affected by phosphorus deficiency stress, the above- and below-ground biomasses were well-maintained through a series of physiological regulation. (3) The combined effect of severe drought and phosphorus deficiency resulted in a greater negative impact on photosynthesis and leaf nitrogen phosphorus ratio in *Carex breviculmis*. (4) Among the anatomical traits, the lower epidermal cells and stomatal width were more special. They showed close correlations with other types of traits such as biomass allocation characteristics, photosynthetic parameter, chemical elements, and specific leaf area.

These research results to some extent reveal the ecological potential of *Carex breviculmis* in the stress environment. Some of strategic features, such as maintenance of biomass under low phosphorus stress, enhancing the relative investment and use efficiency under drought stress, are conducive to plant survival. These stress-tolerance strategies of *Carex breviculmis* may allow it to be potentially promoted in areas with phosphorus deficiency but moderate drought, or in areas with more ideal conditions, laying the foundation for generating habitats with certain biomass and ecological service capacity. These strategies can also be used as a reference for the adaptive capacity of other plants.

However, several issues still need to be further explored in the future. For example, (1) there were some unusual correlations between specific leaf area and some indicators that cannot be fully explained by leaf economics spectrum. It needs to be explored. (2) The molecular regulatory mechanisms of traits in response to stress need further study. Particularly, what biological processes occur under low phosphorus stress that made biomass not significantly affected. (3) Possible soil microbial communities, interspecific interactions and other factors were neglected in this pot experiments. Further field experiments are needed to verify the potential of *Carex breviculmis* for application in natural environments. In summary, the response strategies, mechanisms, and ecological application potential of plants under phosphorus deficiency and drought stress are worth continuous investment.

## Data availability statement

The raw data supporting the conclusions of this article will be made available by the authors, without undue reservation.

## Author contributions

SJ, WL, RF and WJ, designed the experiment, and wrote the manuscript. SJ, YT, SB, XW and YH performed data collection, analysis and interpretation. All authors contributed to the article and approved the submitted version.
