# Supplementary material for: Response of Carex breviculmis to phosphorus deficiency and drought stress
Source: Front Plant Sci. 2023 Jul 11;14:1203924. doi: 10.3389/fpls.2023.1203924 (PMC10366378; doi:10.3389/fpls.2023.1203924)
Supplement: Supplementary file 1 [file Table_1.docx]

Supplementary Material

# Supplementary Tables

**Supplementary Table1.** Summary table of RDA.

| Statistic | Axis 1 | Axis 2 |
| --- | --- | --- |
| Eigenvalues | 0.1560 | 0.0774 |
| Explained variation (cumulative) | 15.60 | 23.34 |
| Pseudo-canonical correlation | 0.9135 | 0.8474 |
| Explained fitted variation (cumulative) | 66.83 | 100.00 |

**Supplementary Table2.** Forward selection results of RDA

| Name | Explains % | Contribution % | pseudo-F | P |
| --- | --- | --- | --- | --- |
| Phosphorus deficiency level | 12.5 | 53.5 | 3.1 | 0.004 |
| Drought level | 10.8 | 46.5 | 3.0 | 0.002 |
